# Cost-utility analysis of the Circle of Security-Parenting programme to reduce perinatal psychopathology in birthing parents in England

**DOI:** 10.1136/bmjopen-2025-105124

**Published:** 2026-02-23

**Authors:** Jiunn Wang, Camilla Rosan, Kim Alyousefi-van Dijk, Zoe Darwin, Daphne Babalis, Victoria Cornelius, Ed Waddingham, Lani Richards, Stephen Pilling, Pasco Fearon, Peter Fonagy, Elena Pizzo

**Affiliations:** 1 Department of Primary Care and Population Health, University College London, London, UK; 2 Department of Clinical, Educational and Health Psychology, University College London, London, UK; 3 Anna Freud Centre, London, UK; 4 School of Human and Health Sciences, University of Huddersfield, Huddersfield, UK; 5 Imperial Clinical Trials Unit, Imperial College London, London, UK; 6 Imperial College London School of Public Health, London, UK; 7 Centre for Child, Adolescent and Family Research, Cambridge University, Cambridge, UK; 8 Division of Psychology and Language Sciences, University College London, London, UK

**Keywords:** Parents, Clinical Trial, Health Care Costs, HEALTH ECONOMICS, MENTAL HEALTH

## Abstract

**Objectives:**

The Circle of Security-Parenting (COS-P) group intervention has demonstrated efficacy in reducing maternal perinatal mental health difficulty (PMHD) symptoms in some contexts. The Circle of Security Intervention (COSI) study, a multisite, individually randomised, single-blind, parallel-arm controlled trial, was conducted in England to assess the clinical effectiveness of COS-P in reducing perinatal psychopathology, parenting and infant development, as well as its acceptability among the National Health Service (NHS) participants and staff. The main aim of this work is to estimate the cost-utility of COS-P plus treatment as usual (TAU) relative to TAU among mothers and birthing parents receiving NHS perinatal mental health services (PMHS) in England.

**Design:**

A within-trial economic evaluation was performed comparing COS-P plus TAU with TAU alone, using data from the COSI trial, which employed a 2:1 randomisation ratio. Analyses were conducted from both NHS and personal social services (PSS) and societal perspectives. A 12-month time horizon was used, consistent with the final trial follow-up.

**Setting:**

Secondary care NHS perinatal health services across multiple centres in England.

**Participants:**

A total of 371 mothers and birthing parents with PMHD were randomised and had complete economic outcome data; 248 received COS-P plus TAU and 123 received TAU alone. Participants were eligible if they were receiving NHS PMHS; exclusion criteria were defined in the trial protocol.

**Interventions:**

Participants in the intervention arm received the COS-P group programme in addition to TAU. The control group received TAU alone.

**Primary and secondary outcome measures:**

The primary economic outcome was quality-adjusted life years (QALYs) over 12 months, derived from the 5-level EuroQol five-dimensional (EQ-5D-5L) questionnaire - responses. Costs were estimated from NHS and PSS as well as societal perspectives, including healthcare utilisation and productivity losses due to work absence.

**Results:**

Compared with TAU, COS-P was associated with higher costs from both NHS and PSS (£180.58; 95% CI −£1075 to £1436) and societal (£72.94; 95% CI −£1473 to £1619) perspectives. COS-P was marginally less effective in terms of QALYs (−0.01; 95% CI −0.06 to 0.05). Probabilistic sensitivity analyses indicated substantial uncertainty around cost and effectiveness estimates.

**Conclusions:**

On average, COS-P was associated with higher costs and did not demonstrate improvements in health-related quality of life compared with TAU alone. Given the uncertainty surrounding the estimates, further research is warranted to explore potential longer term economic and clinical impacts of COS-P in perinatal mental health settings.

**Trial registration number:**

SRCTN18308962.

**STRENGTHS AND LIMITATIONS OF THIS STUDY**

- The analysis was conducted within a large multicentre randomised controlled trial in England using robust and detailed data on participants’ sociodemographic characteristics, resource utilisation and utility measures at multiple time points.
- Analyses were conducted from multiple economic perspectives and validated through rigorous sensitivity analyses, strengthening confidence in the findings.
- Resource use data were collected retrospectively, potentially introducing recall bias.
- There might be uncertainty and variability in the assessment of costs of services and National Health Service resource use and differences between groups in the actual reasons for healthcare use might have influenced cost estimations, although this potential bias was mitigated through extensive sensitivity analysis.
- It is possible that the costs estimated for Circle of Security-Parenting delivery were underestimated, given the information available in group forms and the comments provided by practitioners concerning resource implications.

## Introduction

 Perinatal mental health difficulties (PMHD) encompass mental health difficulties occurring during pregnancy and the first year post-childbirth. Up to 27% of mothers and birthing parents in England experience PMHD.^1^ Untreated PMHD can lead to substantial, lasting consequences for mothers and birthing parents, their children and the broader family. The economic burden associated with PMHD is considerable, estimated at around £10 000 per birth^2 3^ and £8.1 billion per birth cohort.^4^ The National Health Service (NHS) in England provides specialist perinatal mental health services (PMHS) in every area of the country comprising varied care pathways, including preconception advice, specialist psychiatric assessments, care coordination and both psychosocial and psychological treatments from a multidisciplinary team of social workers, nursery nurses, occupational therapists and psychologists.^5^ However, the evidence base supporting these interventions during the perinatal period is inconsistent, with notable gaps.^6^ Emerging evidence indicates that some group-based psychological interventions have demonstrated preliminary effectiveness and are increasingly adopted within NHS community PMHS. One such intervention is Circle of Security-Parenting (COS-P), a brief group intervention rooted in attachment theory, and drawing on psychoeducational, cognitive–behavioural and psychodynamic approaches.^7^ Each session features video clips depicting mother-child interactions, accompanied by reflective discussions on parental experiences and the influence of their upbringing on parenting styles. Although a recent review suggests COS-P may enhance parent-child relationships,^8^ the quality of existing efficacy studies has generally been poor, with no rigorous evaluations to date, and none specifically in the UK nor with perinatal and/or severe mental health samples.^9^ Consequently, the Circle of Security Intervention (COSI), a multisite, individually randomised, single-blind, parallel-arm controlled trial, was conducted in England to assess the clinical effectiveness of COS-P in reducing parental psychopathology, parenting and infant development, as well as its acceptability among NHS participants and staff. Participants were recruited from 10 NHS PMHS in the COSI trial and were randomised to receive either treatment as usual (TAU) alone or COS-P alongside TAU. This study used data from the COSI trial to evaluate the short-term cost-effectiveness of COS-P relative to TAU.

## Materials and methods

### COSI trial and sample

The COSI trial^10^ is a multisite, individually randomised, single-blind, parallel-arm controlled trial with an embedded internal pilot, which recruited 386 participants. A 2:1 randomisation was used in the trial. Out of 386 participants, 15 withdrew prior to randomisation, resulting in a final sample of 371 for analysis. Among these, 123 were allocated to the TAU arm, and 248 to the COS-P alongside the TAU arm. Participants were birthing parents receiving care from participating community PMHS. Eligibility criteria included clinical-level psychopathology (defined by an average Clinical Outcomes in Routine Evaluation-Outcome Measure (CORE-OM) score of 1.1 or more and bonding difficulties indicated by a total Postpartum Bonding Questionnaire (PBQ) score of 12 or more). Detailed information on the trial protocol, clinical effectiveness and acceptability outcomes among parents and practitioners can be found elsewhere.^10 11^

### Intervention and control

Participants were randomised to either the intervention arm, comprising COS-P plus TAU delivered within NHS PMHS, or the control arm receiving TAU alone in PMHS.

#### COS-P programme (intervention)

COS-P is a brief group intervention aimed at promoting parent-child attachment and caregiver sensitivity, while also enhancing social support and peer connections among parents. Delivered by trained and supervised NHS clinicians (typically clinical or counselling psychologists at doctoral level, band 8; supported by a cofacilitator at a similar or a lower band who has not received COS-P training) to groups of four to six parents, the programme comprises eight treatment modules over 10 weekly 90 min sessions, with at least two sessions (the first and one additional session) delivered in person whenever possible, and all others remotely. During sessions, parents view and discuss video clips of parent-child interactions addressing themes such as attachment, caregiving struggles and reflective parenting. A summary of activities delivered within the COS-P programme is provided in table 1.

**Table 1:** Cost of the COS-P intervention

	Length (min)	Staff	Cost	Assumptions and sources
Individual pre-meeting (in person/phone)	30	1 cofacilitator psychologist (bands 3–8)*	£17.74	(Personal Social Services Research Unit, 2024)^25^
1st group intervention (in person)	90	1 facilitator clinical psychologist (band 8) 1 cofacilitator psychologist (bands 3–8)*	£32.24	(Personal Social Services Research Unit, 2024)^25^
2nd–10th group intervention (in person/online)	90	1 facilitator clinical psychologist (band 8) 1 cofacilitator psychologist (bands 3–8)*	£290.17	Assuming the same costs for both in-person and online sessions as they are based on staff salary (PSSRU, 2024)^25^
Catch-up session for those who missed 1/2 sessions	5	1 cofacilitator psychologist (bands 3–8)*	£2.96	Assuming all participants have one missing session (Personal Social Services Research Unit, 2024)^25^
Total intervention cost			£343.10	

Source: internal data and Personal Social Services Research Unit 2024.^25^

* The cost of the cofacilitator is based on the weighted average salary of cofacilitators in the trial data.

COS-P, Circle of Security-Parenting.

#### TAU (comparator)

TAU represents the standard support provided by NHS PMHS as defined by the national service specification.^5^ Typically, TAU involves psychological support delivered by NHS clinicians at band 7 or 8. Examples of TAU service provision across six of the 10 participating trial sites are summarised in table 2. TAU at each of the 10 recruitment sites remained unchanged by participants’ involvement in the trial and was consistent across study groups. TAU was based on a national service specification, providing multidisciplinary, needs-based care, including pharmacological interventions, parent-infant relational interventions, psychological therapies and psychosocial support.^12^

**Table 2:** Cost of the TAU intervention

Site	Item	Staff band	Average time	Frequency	Staff (n)	Cost	Total cost	Weighted average cost
1	Psychosocial assessment/mental health review	4	60	1	1	£36.00	£762.00	£1942.10
	Psychosocial assessment/mental health review	6	60	3	1	£159.00		
	Psychosocial assessment/mental health review	7	60	1	1	£63.00		
	Parent-infant-focused psychological intervention	7	60	8	1	£504.00		
2	Peer support	4	30	1	1	£18.00	£1935.87	
	Psychosocial assessment/mental health review	6	30	7	1	£185.50		
	Mental health-focused psychological intervention (group)	7	120	2	3	£756.00		
	Parent-infant-focused psychological intervention	8a	60	7	1	£504.00		
	Psychiatry review	Psychiatric consultant	45	4	1	£429.00		
	Parent-infant-focused support	3	60	3	1	£43.37		
3	Parent-infant-focused support	4	60	6	2	£432.00	£3767.00	
	Mental health-focused psychological intervention (group)	6	60	25	1	£1325.00		
	Mental health-focused psychological intervention (group)	8a	60	16	1	£1152.00		
	Psychiatry review	Psychiatric consultant	30	12	1	£858.00		
4–6*	Psychosocial assessment/mental health review	6	60	13	1	£689.00	£1729.25	
	Psychosocial assessment/mental health review	7	60	2	1	£126.00		
	Mental health-focused psychological intervention (group)	8a	90	1	1	£108.00		
	Mental health-focused psychological intervention (group)	4	90	1	1	£54.00		
	Parent-infant-focused support	4	60	6	1	£216.00		
	Psychiatry review	Psychiatric consultant	45	5	1	£536.25		

Source: internal data and Personal Social Services Research Unit 2024.^25^

* Data from sites 4–6 were summarised as mean values instead of being reported separately.

TAU, treatment as usual.

### Data

Data were collected at baseline and at 3, 7 and 12-month follow-ups.^10^ The primary clinical outcome of psychopathology was measured using the CORE-OM.^6^ Secondary outcomes included Difficulties in Emotion Regulation Scale (DERS),^13^ PBQ,^14^ Ages and Stages Questionnaire (ASQ),^15^ Ages and Stages Questionnaire: Social-Emotional,^16^ the National Institute of Child Health and Human Development (NICHD) sensitivity scales,^17^ adverse events, healthcare utilisation captured via the Client Service Receipt Inventory (CSRI) Questionnaire^18^ and health-related quality of life assessed using the 5-level EuroQol five-dimensional (EQ-5D-5L) questionnaire.^19 20^

### Patient and public involvement

Patients and the public were involved in the design, conduct, reporting and dissemination of this study. This included a diverse panel of women and birthing people with personal experience of PMHD and/or attending a PMHS who were a core part of the study delivery team.

### Overview of economic evaluation

Although the (clinical) effectiveness and acceptability of COS-P have previously been investigated in some contexts,^9 21^ its cost-effectiveness in a PMHS remains underexplored. In this study, we conducted a within-trial cost-utility analysis to assess the costs and outcomes of COS-P compared with TAU. Outcomes were measured using quality-adjusted life years (QALYs), consistent with the National Institute for Health and Care Excellence (NICE) recommendations.^22^ Cost-utility was expressed as the incremental cost per additional QALY gained. The primary (base case) analysis adopted an NHS and personal social services (PSS) perspective.^22^ Healthcare resource use was assessed using trial data, with unit costs derived from UK standard published sources.^23^,^25^ Costs were expressed in 2023 British pounds and inflated where appropriate.^26^ A 12-month time horizon was employed, corresponding with the trial’s primary follow-up period, which was sufficient to capture relevant cost and outcome differences. Given this short duration, no extrapolation or discounting of costs and outcomes was applied. In addition to the base case NHS perspective, an alternative analysis incorporated a broader societal perspective, including non-NHS costs.

### Resource use and costs

#### Costs of intervention and comparator

The cost of COS-P was estimated based on trial documentation and expert opinion. Intervention costs included one pre-meeting assessment, one initial in-person group session, nine subsequent group sessions (delivered in person or remotely) and one catch-up session for participants who missed up to two sessions. Table 1 summarises total intervention costs and key assumptions.

Given the variability in TAU across trial sites, average TAU costs were estimated using data from six representative sites (Devon Partnership NHS Trust, Sussex Partnership NHS Foundation Trust, Northamptonshire Healthcare NHS Foundation Trust, Mersey Care NHS Foundation Trust (Mid-Mersey and Central Mersey sites) and Cheshire and Wirral Partnership NHS Foundation Trust). These sites provided information regarding the potential standard of care for patients with PMHD. Their representation has been cross-checked against other sites in terms of workforce capacity and delivery taxonomies, geographical distribution and the sociodemographic distribution of the population they served. To standardise, only activities consistently provided across sites (eg, psychological interventions for mental health) were included. We preferred to get a bespoke retrospective report of the services provided to ensure that more detailed and high-quality data could be collected by a representative sample of services, based on what was actually provided. Table 2 summarises TAU cost components and total cost estimates.

#### Costs of NHS and PSS

Costs of NHS and PSS use per participant (including general practitioner consultations, community and district nursing visits, accident and emergency (A&E) attendance, general and psychiatric hospital admissions, outpatient appointments and medication usage) were calculated by multiplying recorded service utilisation by published unit costs.^23^,^25^ Resource utilisation data were collected retrospectively using an adapted version of the CSRI. For analyses adopting the societal perspective, additional costs included private healthcare expenses (out-of-pocket payments) and productivity losses due to time off work to attend intervention sessions, calculated using average daily salary data from the CSRI.

### Effectiveness, utility and QALYs

Effectiveness was measured using validated questionnaires, including PBQ, DERS, ASQ and CORE-OM, as detailed in the primary trial publication.^11 27^ Generic health-related quality of life was assessed via the EQ-5D-5L questionnaire^19^ administered at baseline and at 3, 7 and 12-month follow-ups. EQ-5D-5L responses were converted into utility scores using UK population-specific valuation weights.^28 29^ Utility scores range from 0 (death) to 1 (full health), with negative values representing health states considered worse than death. No death occurred with participants in the trial. Participant-specific utility profiles were generated by linear interpolation between utility values recorded at each measurement point. QALYs were computed as the area under each participant’s utility profile from baseline to 12-month follow-up.

### Statistical analysis

Mean differences in costs and QALYs between the COS-P and TAU groups were calculated. Uncertainty surrounding these differences was estimated using bootstrapping with 1000 replicates.^30^ Incremental cost-effectiveness ratios (ICERs) were computed as the incremental cost per additional QALY gained. Net monetary benefits (NMBs) were derived by multiplying mean QALYs per participant by NICE-recommended willingness-to-pay thresholds (£20 000–£30 000 per QALY gained) minus mean costs per participant. Incremental NMBs (INMBs) were calculated similarly, based on differences in mean QALYs and costs between COS-P and TAU. A positive INMB favoured COS-P, while a negative value indicated preference for TAU on cost-effectiveness grounds.

### Missing data

Missing data for NHS and private costs, utility values at each time point and total QALYs were jointly imputed using multiple imputation by chained equations. Age, sociodemographic variables and clinical variables were included as explanatory variables. 50 imputed datasets were generated to reflect the proportion of incomplete cases,^30 31^ and results were pooled according to Rubin’s rules.^32^

### Sensitivity analysis

Probabilistic sensitivity analysis was conducted to evaluate robustness by assigning statistical distributions to model parameters: gamma distributions were used for costs, and beta distributions for utilities. Parameters in the distributions were based on the statistics such as means and SDs derived from the imputed data. Uncertainty was captured using bootstrapping with 1000 replicates per imputed dataset, and estimates were combined using Rubin’s rule. Cost-effectiveness acceptability curves^33^ were generated, illustrating the probability that COS-P was cost-effective relative to TAU across the NICE-recommended threshold range (£20 000–£30 000 per QALY). Probabilities of cost-effectiveness at thresholds of £20 000 and £30 000 were reported. All analyses were conducted from both NHS/PSS and societal perspectives.

## Results

In our dataset, 371 participants had been randomised in a 2:1 allocation ratio to either TAU (n=123) or COS-P alongside TAU (n=248). Baseline characteristics of the participants in the two groups are presented in online supplemental table A1. The mean age across both groups was 30.79 years (SD 5.44). A total of 329 (88.68%) participants reported their ethnicity as white; nine (2.43%) were of mixed ethnicity; three (0.81%) were Asian; and one (0.27%) was black. The distribution of demographic and clinical variables was similar across two groups. Baseline health service use costs (TAU £296.63, COS-P £271.18), costs of productivity loss (TAU £47.44, COS-P £36.18) and EQ-5D-5L scores (TAU 0.60, COS-P 0.62) were comparable.

Baseline assessments for cost and utilities were completed by 334 participants, with follow-up EQ-5D data obtained from 274 (3 months), 233 (7 months) and 226 (12 months), and follow-up resource use data from 276 (3 months), 237 (7 months) and 229 (12 months). Complete health economics data were available for 71 in the TAU arm and 119 participants in the COS-P alongside the TAU arm. Follow-up completion rates are 75.6% (n=93) in TAU and 72.2% (n=179) in COS-P alongside TAU at 3 months, and 63.4% (n=78) in TAU and 58.9% (n=146) in COS-P alongside TAU at 12 months.

### Costs of the intervention and TAU

The estimated per-recipient cost of delivering COS-P was £343 (table 1). The estimated average cost for TAU, accounting for variability across sites, was £1942 (table 2). As participants in the intervention arm received COS-P alongside TAU, the combined average cost was £2285.

### NHS costs of health resource use and costs

Health resource use and costs for the complete cases and unit cost data are reported in online supplemental tables A2–A4. The NHS cost trajectory does not indicate major differences between the two arms. Average NHS cost at 12 months was lower for TAU participants (£309.36) compared with COS-P participants (£383.83). However, from a societal perspective, TAU participants incurred additional private healthcare expenses and productivity losses, averaging £31.27 higher compared with the COS-P group (online supplemental table A4), but these differences were offset by the higher costs to the NHS at 12 months.

### Utilities and QALYs

EQ-5D-5L utility scores at baseline were comparable (COS-P 0.62, TAU 0.60); at 3 months, utilities slightly favoured COS-P (COS-P 0.65 vs TAU 0.63), but this reversed at 7 months (COS-P 0.67 vs TAU 0.68) and at 12 months (COS-P 0.68 vs TAU 0.70). The full trajectory of utility scores is presented in table 3.

**Table 3:** Mean utilities at baseline and 3, 7 and 12 months, based on observed data

Group	Baseline		3 months		7 months		12 months	
	n	Mean (SD)	n	Mean (SD)	n	Mean (SD)	n	Mean (SD)
TAU	116	0.60 (0.25)	94	0.63 (0.26)	87	0.68 (0.26)	79	0.70 (0.23)
COS-P	223	0.62 (0.23)	180	0.65 (0.23)	146	0.67 (0.24)	147	0.68 (0.22)

COS-P, Circle of Security-Parenting; TAU, treatment as usual.

### Cost-utility

Cost-utility was assessed using ICERs, calculated as the incremental cost per QALY gained by COS-P compared with TAU. From an NHS perspective, COS-P generated a cost increment of £180.58 per participant (95% CI −£1075 to £1436) compared with TAU, with negligible differences in QALYs (−0.0068; 95% CI −0.06 to 0.05). Although the ICER value (−£26 447 per QALY) was calculated, it has limited interpretability because the base case scenario lies within the cost-effectiveness plane’s top-left quadrant, indicating cost increments accompanied by lower effectiveness. NMBs were consistently higher for TAU than COS-P at both lower (£7295 for TAU vs £6978 for COS-P) and upper (£14 094 for TAU vs £13 709 for COS-P) NICE thresholds, reinforcing the finding that COS-P is not cost-effective (table 4).

**Table 4:** Deterministic results: average costs, QALYs, ICER and NMBs of COS-P versus TAU (NHS perspective), based on imputed data

	TAU	COS-P
Intervention cost	£1942.10	£2285.21
Health service cost	£4361.43	£4198.90
Total cost	£6303.53	£6484.11
QALYs	0.6799	0.6731
Incremental cost (95% CI)		£180.58 (−£1075 to £1436)
Incremental QALY (95% CI)		−0.0068 (−0.06 to 0.05)
ICER		−£26 447.33
Net monetary benefit (CE threshold: 20 000)	£7294.76	£6977.63
Net monetary benefit (CE threshold: 30 000)	£14 093.91	£13 708.50
Incremental NMB (CE threshold: 20 000)		−£317.13
Incremental NMB (CE threshold: 30 000)		−£385.41

Missing data were imputed using 50 datasets via multiple imputation by chained equations with predicted mean matching. Variances were pooled using Rubin’s rule to derive 95% CIs.

CE, cost-effectiveness; COS-P, Circle of Security-Parenting; ICER, incremental cost-effectiveness ratio; NHS, National Health Service; NMB, net monetary benefit; QALY, quality-adjusted life years; TAU, treatment as usual.

From a societal perspective, when accounting for private healthcare expenses and productivity losses, total average costs were slightly higher for COS-P (£7234) than TAU (£7162), though there was still a high level of uncertainty around these estimates (table 5).

**Table 5:** Deterministic results: average costs, QALYs, ICER and NMBs of COS-P versus TAU (societal perspective), based on imputed data

	TAU	COS-P
Intervention cost	£1942.10	£2285.21
Health service cost	£4402.12	£4319.03
Productivity loss	£817.30	£630.23
Total cost	£7161.52	£7234.46
QALYs	0.6799	0.6731
Incremental cost (95% CI)		£72.94 (−£1473 to £1619)
Incremental QALY (95% CI)		−0.0068 (−0.06 to 0.05)
ICER		−£10 682.32
NMB (CE threshold: 20 000)	£6436.77	£6227.28
NMB (CE threshold: 30 000)	£13 235.92	£12 958.15
Incremental NMB (CE threshold: 20 000)		−£209.49
Incremental NMB (CE threshold: 30 000)		−£277.77

Missing data were imputed using 50 datasets via multiple imputation by chained equations with predicted mean matching. Variances were pooled using Rubin’s rule to derive 95% CIs.

CE, cost-effectiveness; COS-P, Circle of Security-Parenting; ICER, incremental cost-effectiveness ratio; NMB, net monetary benefit; QALY, quality-adjusted life year; TAU, treatment as usual.

### Results of the sensitivity analysis

Probabilistic sensitivity analyses confirmed the deterministic results. From an NHS perspective, COS-P generated an increase in average cost by £182.53 per participant (95% CI −£492 to £857) and a decrease in QALYs (−0.01; 95% CI −0.04 to 0.02). NMBs were higher for TAU at both the lower threshold (£7298 vs £6978 for TAU and COS-P, respectively) and the upper threshold (£14 098 vs £13 709 for TAU and COS-P, respectively) (table 6).

From a societal perspective, COS-P incurred an additional £72.83 per participant and a decrease in average QALYs (by −0.01) compared with TAU, with uncertainty around the estimates (table 7).

**Table 6:** Probabilistic results: average costs, QALYs, ICER and NMBs of COS-P (NHS perspective), based on imputed data

	TAU	COS-P
Intervention cost	£1942.11	£2285.21
Health service cost	£4360.31	£4199.74
Total cost	£6302.42	£6484.95
QALY	0.6800	0.6731
Incremental cost (95% CI)		£182.53 (−£492 to £857)
Incremental QALY (95% CI)		−0.0069 (−0.04 to 0.02)
ICER		−£26 539.69
NMB (CE threshold: 20 000)	7297.72	6977.63
NMB (CE threshold: 30 000)	14 097.79	13 708.93
Incremental NMB (CE threshold: 20 000)		−320.09
Incremental NMB (CE threshold: 30 000)		−388.86

Missing data were imputed using 50 datasets via multiple imputation by chained equations with predicted mean matching. Variances were pooled using Rubin’s rule to derive 95% CIs.

CE, cost-effectiveness; COS-P, Circle of Security-Parenting; ICER, incremental cost-effectiveness ratio; NHS, National Health Service; NMB, net monetary benefit; QALY, quality-adjusted life year; TAU, treatment as usual.

**Table 7:** Probabilistic results: average costs, QALYs, ICER and NMBs of COS-P (societal perspective), based on imputed data

	TAU	COS-P
Intervention cost	£1942.11	£2285.21
Health service cost	£4401.27	£4319.62
Productivity loss	£818.37	£629.74
Total cost	£7161.75	£7234.58
QALYs	0.6800	0.6731
Incremental cost (95% CI)		£72.83 (−£660 to £806)
Incremental QALY (95% CI)		−0.0069 (−0.04 to 0.02)
ICER		−£10 588.73
NMB (CE threshold: 20 000)	£6438.39	£6228.01
NMB (CE threshold: 30 000)	£13 238.46	£12 959.30
Incremental NMB (CE threshold: 20 000)		−£210.38
Incremental NMB (CE threshold: 30 000)		−£279.16

Missing data were imputed using 50 datasets via multiple imputation by chained equations with predicted mean matching. Variances were pooled using Rubin’s rule to derive 95% CIs.

CE, cost-effectiveness; COS-P, Circle of Security-Parenting; ICER, incremental cost-effectiveness ratio; NMB, net monetary benefit; QALY, quality-adjusted life year; TAU, treatment as usual.

**Figure 1:**
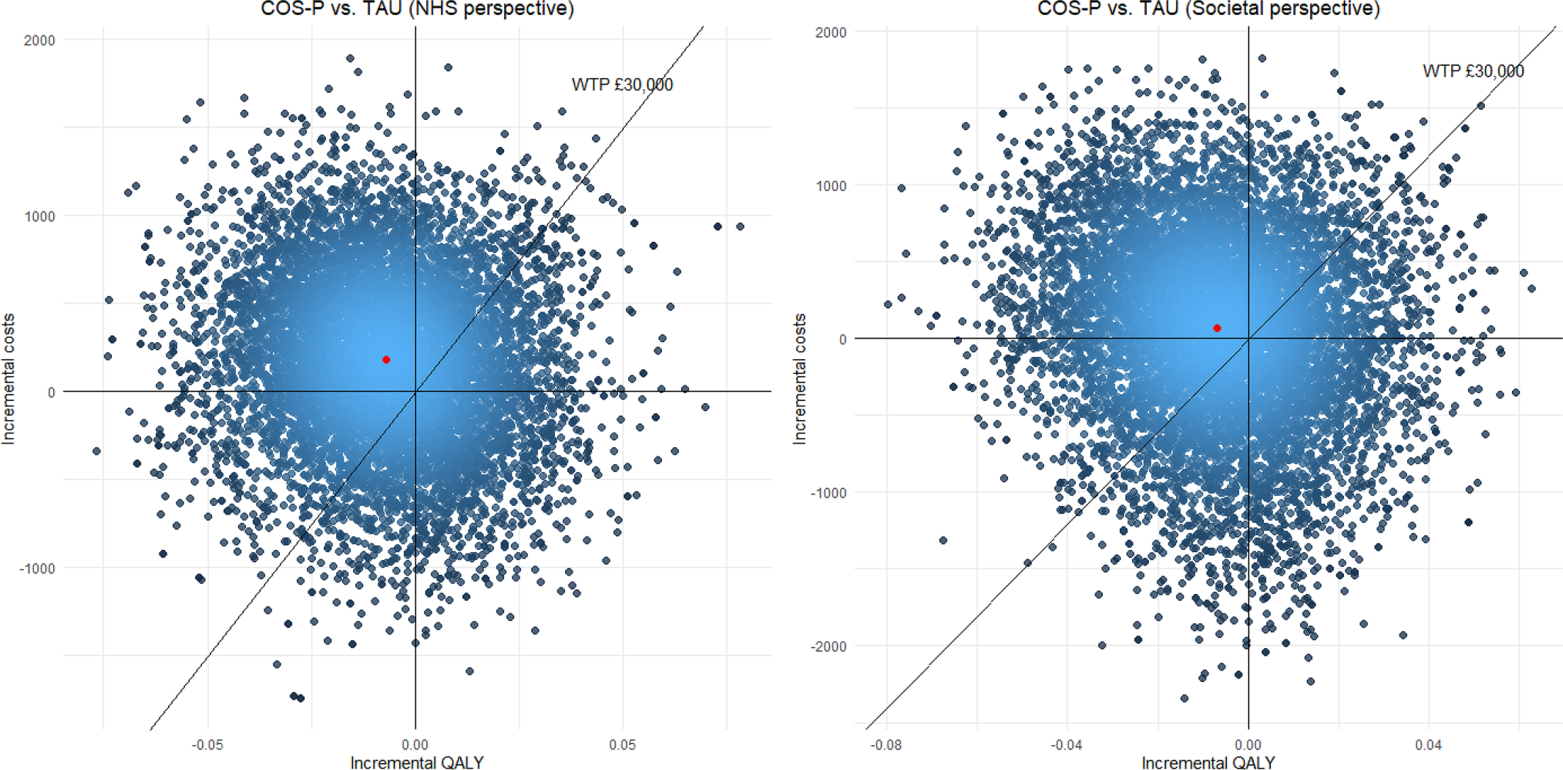
Monte Carlo simulations of incremental cost per QALY of COS-P. COS-P, Circle of Security-Parenting; NHS, National Health Service; QALY, quality-adjusted life year; TAU, treatment as usual; WTP, willingness to pay.

**Figure 2:**
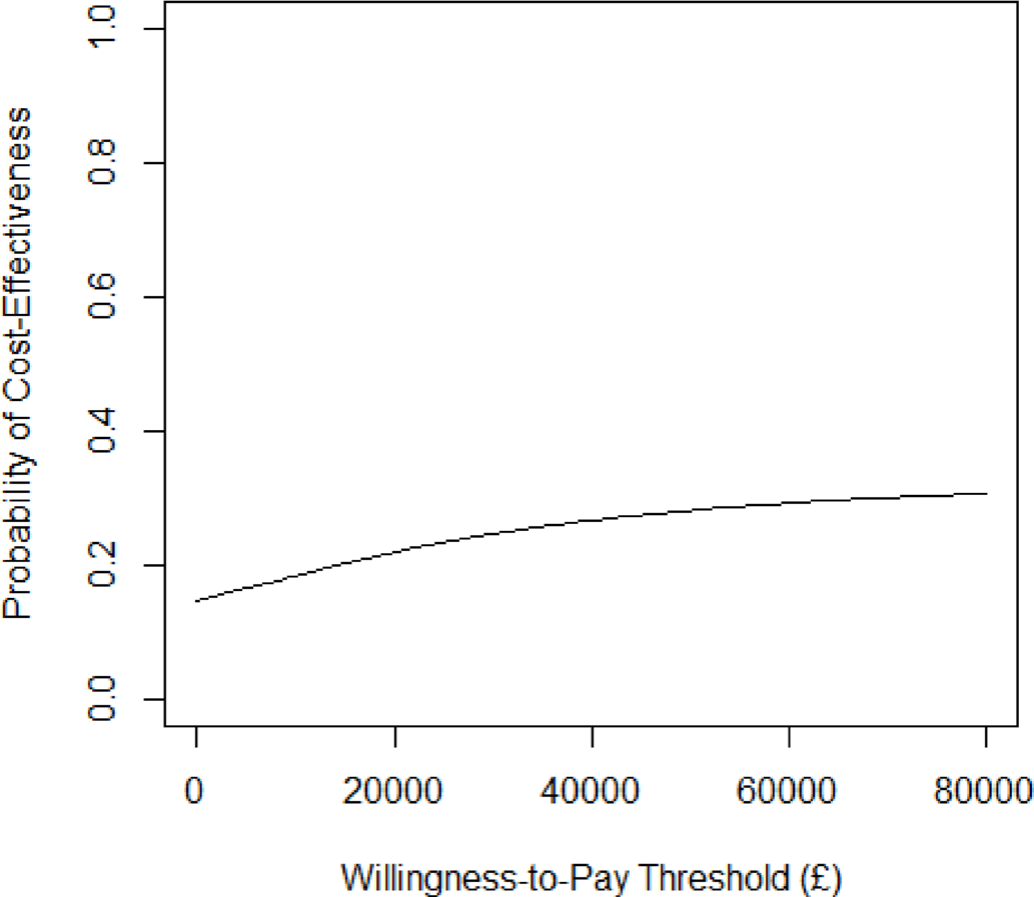
Cost-effectiveness acceptability curve showing the probability that COS-P versus TAU is cost-effective at different values of the maximum willingness to pay for a QALY. The probability that TAU is cost-effective is 1 minus the probability that COS-P is cost-effective at each value of the maximum willingness to pay for a QALY. COS-P, Circle of Security-Parenting; QALY, quality-adjusted life year; TAU, treatment as usual.

Probabilistic sensitivity analyses and Monte Carlo simulations presented in figure 1 illustrate the high uncertainty surrounding ICER estimates, with data points distributed across all four quadrants of the cost-effectiveness plane. The cost-effectiveness acceptability curve (figure 2) further demonstrates that the probability of COS-P being cost-effective compared with TAU across willingness-to-pay thresholds for a QALY is effectively negligible.

A one-way sensitivity analysis examining how different elements affect the health economic results is provided in online supplemental table A5.

## Discussion

The COSI trial found no significant improvement on perinatal psychopathology, or any secondary outcomes, by adding COS-P to TAU in NHS PMHS. Although the intervention was favoured for some outcomes at some time points, the effects were not statistically significant. Our economic evaluation indicated that, on average, COS-P alongside TAU was associated with higher NHS costs and lower QALYs compared with TAU alone, although there was a high level of uncertainty around these point estimates. From a societal perspective, COS-P was associated with lower private healthcare costs and reduced productivity loss, but these savings were offset by higher NHS costs. Hence, the intervention was not considered cost-effective from a societal perspective. Extensive sensitivity analyses supported these conclusions, consistently showing COS-P to be not cost-effective at NICE’s recommended willingness-to-pay thresholds of £20 000–£30 000 per QALY gained.

While a previous cost-benefit analysis conducted in the USA suggested COS-P might yield positive NMBs after 20 years,^34^ direct comparisons are limited. The US-based study differs substantially from ours, using monetary valuations of benefits rather than QALYs, reflecting significant differences between the UK NHS and the US healthcare system. Moreover, the US intervention was longer, and the analysis employed a substantially extended time horizon.

To our knowledge, this is the first cost-utility analysis of COS-P conducted within a large multicentre randomised controlled trial in England. A key strength of this study is the robust and detailed data on participants’ sociodemographic characteristics, resource utilisation and utility measures at multiple time points. Additionally, analyses were conducted from multiple economic perspectives and validated through rigorous sensitivity analyses, strengthening confidence in the findings.

Our study has several limitations. First, resource use data were collected retrospectively (covering the previous 3–5 months), potentially introducing recall bias; however, such bias is unlikely to differ systematically between COS-P and TAU groups. Second, due to incomplete details regarding reasons for NHS service utilisation, average unit costs for A&E visits, hospital admissions and outpatient attendances were applied. Differences between groups in the actual reasons for healthcare use might have influenced cost estimations, although this potential bias was mitigated through extensive probabilistic sensitivity analysis. Similarly, missing information necessitated assumptions regarding medication types, dosages and associated costs. Third, substantial variability exists in the types of services provided to parents experiencing PMHD across NHS sites. Consequently, we estimated an average cost for TAU based on data from six representative trial sites, which might not fully reflect the breadth of TAU services provided across England. Additionally, there is potential for double counting of TAU costs if participants reported the same services within the CSRI data. However, given that COS-P was delivered alongside TAU, this would not materially disadvantage TAU in comparative terms. Fourth, our costing may not fully capture the variability of the intervention. Depending on the participants and the practitioners, the number and duration of catch-up sessions as well as the length of pre-meetings could vary from case to case. In addition, extra tasks related to session preparation and postsession documentation were not recorded in our data. Nevertheless, our probabilistic sensitivity analysis partially accounts for this variability, with the results supporting our deterministic results. Fifth, the EQ-5D-5L questionnaire might not be the best instrument to capture postnatal mental health impacts on quality of life, because it does not directly assess aspects such as fatigue, cognitive issues, social isolation, spirituality and other emotional states beyond ‘anxiety and depression’ that are relevant to birthing parents, and it has limited sensitivity to change in this population.

Finally, our analysis employed a 12-month time horizon aligned with primary trial follow-up. While adopting a shorter horizon might have highlighted immediate benefits observed at 3 months, or a longer horizon could have captured delayed impacts, neither approach would likely alter conclusions, given the lack of significant differences at 12 months. It is possible that the costs estimated for COS-P delivery are underestimated, given the information available in group forms and comments provided by practitioners concerning the resource implications. However, if the costs are greater than those estimated here, it would not challenge the conclusion of this analysis, that is, that the probability of COS-P being cost-effective within the UK NHS context is low.

In addition to the cost-effectiveness results reported here, the COSI trial has found no additional clinical benefit from COS-P as compared with TAU alone.^26^ Therefore, we do not recommend the use of COS-P in NHS PMHS in the format used here.

## Conclusions

Overall, we do not find evidence supporting the cost-effectiveness of COS-P for improving perinatal psychopathology among parents experiencing PMHD in NHS PMHS in England. Further research is required to establish more consistent and standardised PMHS interventions addressing mental health difficulties during pregnancy and post partum.

## Supplementary material

10.1136/bmjopen-2025-105124 online supplemental file 1

## Data Availability

Data are available upon reasonable request.
