# Supplementary material for: Cost-utility analysis of the Circle of Security-Parenting programme to reduce perinatal psychopathology in birthing parents in England
Source: BMJ Open. 2026 Feb 23;16(2):e105124. doi: 10.1136/bmjopen-2025-105124 (PMC12931550; doi:10.1136/bmjopen-2025-105124)
Supplement: online supplemental file 1 [file bmjopen-16-2-s001.docx]

**Supplementary materials**

**Table A1. Baseline Characteristics**

|  | **TAU (n = 123)** | **COS-P (n = 248)** | **Total (n = 371)** |
| --- | --- | --- | --- |
| Age | 31.05 (5.61) | 30.67 (5.36) | 30.79 (5.44) |
| Ethnicity |  |  |  |
| White | 113 (91.87%) | 216 (87.10%) | 329 (88.68%) |
| Black | 0 (0.00%) | 1 (0.40%) | 1 (0.27%) |
| Mixed | 3 (2.44%) | 6 (2.42%) | 9 (2.43%) |
| Asian | 1 (0.81%) | 2 (0.81%) | 3 (0.81%) |
| Other | 1 (0.81%) | 1 (0.40%) | 2 (0.54%) |
| Missing | 5 (4.07%) | 22 (8.87%) | 27 (7.28%) |
| Current living situation |  |  |  |
| Living alone | 10 (8.13%) | 21 (8.47%) | 31 (8.36%) |
| Living with partner | 96 (78.05%) | 188 (75.81%) | 284 (76.55%) |
| Living with other relatives | 11 (8.94%) | 13 (5.24%) | 24 (6.47%) |
| Living with others | 1 (0.81%) | 4 (1.61%) | 5 (1.35%) |
| Prefer not to say | 0 (0.00%) | 1 (0.40%) | 1 (0.27%) |
| Missing | 5 (4.07%) | 21 (8.47%) | 26 (7.01%) |
| Education |  |  |  |
| Primary or less | 0 (0.00%) | 4 (1.61%) | 4 (1.08%) |
| Secondary | 9 (7.32%) | 17 (6.85%) | 26 (7.01%) |
| Tertiary or further education | 36 (29.27%) | 75 (30.24%) | 111 (29.92%) |
| Higher education | 70 (56.91%) | 127 (51.21%) | 197 (53.10%) |
| Other general education | 2 (1.63%) | 2 (0.81%) | 4 (1.08%) |
| Prefer not to say | 0 (0.00%) | 1 (0.40%) | 1 (0.27%) |
| Missing | 6 (4.88%) | 22 (8.87%) | 28 (7.55%) |
| Been pregnant before |  |  |  |
| Yes | 78 (63.41%) | 148 (59.68%) | 226 (60.92%) |
| No | 39 (31.71%) | 82 (33.06%) | 121 (32.61%) |
| Prefer not to say | 2 (1.63%) | 3 (1.21%) | 5 (1.35%) |
| Missing | 4 (3.25%) | 15 (6.05%) | 19 (5.12%) |
| Previous pregnancy loss |  |  |  |
| Yes | 58 (47.15%) | 109 (43.95%) | 167 (45.01%) |
| No | 17 (13.82%) | 37 (14.92%) | 54 (14.56%) |
| Prefer not to say | 3 (2.44%) | 2 (0.81%) | 5 (1.35%) |
| Missing | 45 (36.59%) | 100 (40.32%) | 145 (39.08%) |
| Mental health difficulties leading to community perinatal mental health services referral |  |  |  |
| Depression | 97 (78.86%) | 204 (82.26%) | 301 (81.13%) |
| Anxiety | 101 (82.11%) | 199 (80.24%) | 300 (80.86%) |
| Trauma | 42 (34.15%) | 101 (40.73%) | 143 (38.54%) |
| Personality difficulties | 20 (16.26%) | 36 (14.52%) | 56 (15.09%) |
| Obsessive compulsive disorder | 11 (8.94%) | 32 (12.90%) | 43 (11.59%) |
| Psychosis | 2 (1.63%) | 10 (4.03%) | 12 (3.23%) |
| Bipolar disorder | 5 (4.07%) | 10 (4.03%) | 15 (4.04%) |
| Other | 7 (5.69%) | 13 (5.24%) | 20 (5.39%) |
| Child age (in weeks) | 21.48 (12.95) | 21.40 (12.48) | 21.43 (12.62) |
| Sex of child |  |  |  |
| Female | 62 (50.41%) | 113 (45.56%) | 175 (47.17%) |
| Male | 56 (45.53%) | 121 (48.79%) | 177 (47.71%) |
| Missing | 5 (4.07%) | 14 (5.65%) | 19 (5.12%) |
| Health service use cost (£) |  |  |  |
| NHS | 287.80 (417.61) | 270.35 (501.42) | 276.32 (473.86) |
| Private | 8.84 (50.51) | 0.83 (9.06) | 3.57 (30.60) |
| Total | 296.63 (428.63) | 271.18 (501.09) | 279.89 (477.04) |
| Cost of productivity loss (£) | 47.44 (246.75) | 36.18 (231.26) | 40.00 (236.33) |
| EQ-5D-5L index score | 0.60 (0.25) | 0.62 (0.23) | 0.61 (0.24) |

Data are n (%) or mean (SD)

**Table A2 Unit cost data for healthcare services and staff time**

| **Health service** | **Unit cost** | **Note** | **Source** |
| --- | --- | --- | --- |
| **Intervention** |  |  |  |
| Clinical psychologist Band 8a | £72.00 | Unit cost per hour for community-based scientific and professional Band 8a | PSSRU 2022-2023; p.56 |
| Clinical psychologist Band 8b | £84.00 | Unit cost per hour for community-based scientific and professional Band 8b | PSSRU 2022-2023; p.56 |
| Clinical psychologist Band 7 | £63.00 | Unit cost per hour for community-based scientific and professional Band 7 | PSSRU 2022-2023; p.56 |
| Clinical psychologist Band 6 | £53.00 | Unit cost per hour for community-based scientific and professional Band 6 | PSSRU 2022-2023; p.56 |
| Clinical psychologist Band 4 | £36.00 | Unit cost per hour for community-based scientific and professional Band 4 | PSSRU 2022-2023; p.56 |
| Clinical psychologist Band 3 | £14.46 | Unit cost per hour for NHS professional Band 3, assuming 48 working weeks per year and 37 working hour per week | NHS pay and benefits |
| GP | £49.00 | Unit costs for a GP per surgery consultation lasting 10 minutes | PSSRU 2022-2023; p.64 |
| Community/District Nurse | £10.67 | Cost per hour including qualifications for a nurse at Band 6 is £64. We assume that each consultation lasts 10 mins. | PSSRU 2022-2023; p.61 |
| A&E | £263.00 | National average of emergency care | National Tariff 2023 |
| Walk-in Centre | £80.00 | National average of community health services | National Tariff 2023 |
| Hospital inpatient ward - acute hospital (other than ICU) | £242.00 | National average of regular day and night admissions | National Tariff 2023 |
| ICU admission | £402.82 | E04 Enhanced/Intensive Support Service for Mental Health Care Contact | National Tariff 2023 |
| Hospital inpatient ward - psychiatric hospital | £242.00 | National average of regular day and night admissions | National Tariff 2023 |
| Community hospital ward | £242.00 | National average of regular day and night admissions | National Tariff 2023 |
| Outpatient | £225.00 | National average of outpatient procedures | National Tariff 2023 |
| Day hospital | £225.00 | National average of outpatient procedures | National Tariff 2023 |
| Community care services | £1.05 | Unit cost per minute for community-based scientific and professional Band 7 | PSSRU 2022-2023; p.56 |
| Psychologist | £63.00 | Unit cost per hour for community-based scientific and professional Band 7 | PSSRU 2022-2023; p.56 |
| NHS 111 | £12.65 | Cost of a call to NHS 111 by telephone | Turner et al (2012), inflated to 2023 price |
| Ambulance | £244.00 | National average of ambulance | National Tariff 2023 |
| Dentist | £116.00 | Unit cost per hour for NHS dentist in a providing-performer practice | PSSRU 2022-2023; p.68 |
| Other professionals | £63.00 | Unit cost per hour for community-based scientific and professional Band 7 | PSSRU 2022-2023; p.56 |
| Community mental health care | £61.00 | Unit cost per client visit for local authority own-provision social services day care for adults requiring mental health services | PSSRU 2022-2023; p.15 |
| Psychiatrist | £63.00 | Unit cost per hour for community-based scientific and professional Band 7 | PSSRU 2022-2023; p.56 |
| Physiotherapist | £63.00 | Unit cost per hour for community-based scientific and professional Band 7 | PSSRU 2022-2023; p.56 |
| Ultrasound | £196.99 | MA36Z Transvaginal Ultrasound (regular day or night admissions) | National Tariff 2023 |

**Table A3 Unit cost data for medications**

| **Medication** | **Dosage** | **Unit cost** |
| --- | --- | --- |
| Amitriptyline | 10mg | £0.05 |
| Amitriptyline | 50mg | £0.21 |
| Amitriptyline | 150mg | £0.64 |
| Amitriptyline | 25mg | £0.06 |
| Amlodipine | 10mg | £0.31 |
| Amoxicillin | 500mg | £0.50 |
| Amitriptyline | 5mg | £0.01 |
| Aripiprazole | 15mg | £0.05 |
| Aripiprazole | 5mg | £0.05 |
| Aripiprazole | 10mg | £0.05 |
| Aripiprazole | depot | £220.41 |
| Aripiprazole | 2.5mg | £1.05 |
| Aripiprazole | 80mg | £0.42 |
| Avamys | 27.5mg | £0.05 |
| Beclometasone dipropionate | 200mg | £0.08 |
| Quetiapine | 150mg | £0.82 |
| Quetiapine | 200mg | £0.82 |
| Bisoprolol | 1.25mg | £0.03 |
| Bisoprolol | 2.5mg | £0.02 |
| Budesonide | 200micrograms | £0.14 |
| Budesonide | 9mg | £2.50 |
| Bupropion | 150mg | £0.70 |
| Buspirone | 15mg | £0.70 |
| Buspirone | 5mg | £0.44 |
| Glatiramer | 40mg | £38.55 |
| Carbimazole | 20mg | £2.08 |
| Catalipram | 20mg | £0.02 |
| Desogestrel | 75mg | £0.27 |
| Citalopram | 10mg | £0.44 |
| Cetirizine hydrochloride | 20mg | £0.05 |
| Cetirizine hydrochloride | 10mg | £0.03 |
| Clique | 250micrograms | £0.07 |
| Citalopram | 20mg | £0.52 |
| Citalopram | 40mg | £0.59 |
| Clomipramine | 50mg | £0.50 |
| Clobazam | 10mg | £0.22 |
| Co-codamol | 30mg | £0.04 |
| Codeine | 15mg | £0.05 |
| Codeine | 60mg | £0.06 |
| Codeine | 30mg | £0.06 |
| Colecalciferol | 800unit | £0.17 |
| Methylphenidate | 54mg | £1.42 |
| Methylphenidate | 36mg | £1.42 |
| Cyclizine | 50mg | £0.17 |
| Dexamfetamine sulfate | 20mg | £2.65 |
| Diazepam | 2mg | £0.14 |
| Diazepam | 5mg | £0.23 |
| Dihydrocodeine | 60mg | £0.09 |
| Domperidone | 10mg | £0.07 |
| Doxycycline | 100mg | £0.61 |
| Duloxetine | 30mg | £0.80 |
| Duloxetine | 90mg | £0.47 |
| Duloxetine | 60mg | £0.99 |
| Fluconazole | 50mg | £0.14 |
| Escitalopram | 20mg | £0.90 |
| Galcanezumab | 120mg | £450.00 |
| Empagliflozin | 10mg | £1.31 |
| Escitalopram | 10mg | £0.53 |
| Escitalopram | 5mg | £0.32 |
| Etoricoxib | 90mg | £0.11 |
| Estradiol | 100micrograms | £0.64 |
| Ferrous sulfate | 200mg | £0.07 |
| Ferrous fumarate | 210mg | £0.05 |
| Fexofenadine | 180mg | £0.25 |
| Fluoxetine | 40mg | £0.08 |
| Fluoxetine | 20mg | £0.08 |
| Flucloxacillin | 500mg | £0.08 |
| Fluoxetine | 60mg | £0.09 |
| Flupentixol | 3mg | £0.14 |
| Folic acid | 5mg | £0.07 |
| Folic acid | 400micrograms | £0.02 |
| Fostair | 200micrograms | £0.24 |
| Fusacomb | 50micrograms | £0.36 |
| Gabapentin | 100mg | £0.01 |
| Gabapentin | 300mg | £0.02 |
| Gedarel | 150mg | £0.07 |
| Haloperidol | 1.5mg | £0.18 |
| Certolizumab pegol | 200mg | £357.50 |
| Hydroxocobalamin | 1mg | £2.14 |
| Hydroxychloroquine sulfate | 200mg | £0.04 |
| Lamotrigine | 50mg | £0.03 |
| Lamotrigine | 100mg | £0.04 |
| Lamotrigine | 200mg | £0.06 |
| Lamotrigine | 25mg | £0.28 |
| Lurasidone hydrochloride | 18.5mg | £2.86 |
| Levothyroxine | 100mg | £0.06 |
| Levothyroxine | 50mg | £0.05 |
| Lidocaine | 100mg | £0.66 |
| Lisdexamfetamine | 40mg | £2.24 |
| Loratadine | 10mg | £0.02 |
| Lorazepam | 1mg | £0.24 |
| Losartan | 100mg | £0.58 |
| Mirtazapine | 30mg | £0.15 |
| Mirtazapine | 15mg | £0.16 |
| Mirtazapine | 45mg | £0.18 |
| Metformin | 500mg | £0.09 |
| Methyldopa | 250mg | £0.44 |
| Methylphenidate hydrochloride | 20mg | £0.36 |
| Microgynon | 150mg | £0.04 |
| Minoxidil | 5mg | £0.26 |
| Montelukast | 10mg | £0.96 |
| Tirzepatide | 10mg | £107.00 |
| Naproxen | 500mg | £0.15 |
| Nifedipine | 40mg | £0.48 |
| Nifedipine | 5mg | £0.79 |
| Nitrofurantoin | 100mg | £0.17 |
| Olanzapine | 10mg | £0.05 |
| Olanzapine | 15mg | £0.06 |
| Olanzapine | 5mg | £0.05 |
| Olanzapine | 7.5mg | £0.04 |
| Olanzapine | 2.5mg | £0.04 |
| Omeprazole | 20mg | £0.48 |
| Omeprazole | 40mg | £0.63 |
| Oxybutynin | 2.5mg | £0.02 |
| Paroxetine | 20mg | £0.61 |
| Pizotifen | 1.5mg | £0.06 |
| Propranolol | 10mg | £0.25 |
| Prednisolone | 30mg | £1.04 |
| Pregabalin | 150mg | £0.05 |
| Pregabalin | 75mg | £0.12 |
| Pregabalin | 50mg | £0.07 |
| Progesterone | 200mg | £0.27 |
| Promethazine | 25mg | £0.17 |
| Propranolol | 40mg | £0.03 |
| Propranolol | 80mg | £0.02 |
| Budesonide | 200micrograms | £0.15 |
| Pyridoxine | 20mg | £0.67 |
| Quetiapine | 50mg | £0.49 |
| Quetiapine | 25mg | £0.81 |
| Quetiapine | 100mg | £2.26 |
| Quetiapine | 300mg | £1.24 |
| Ramipril | 2.5mg | £0.24 |
| Risperidone | 1mg | £0.04 |
| Risperidone | 3mg | £0.03 |
| Salbutamol | 100micrograms | £0.03 |
| Sertraline | 50mg | £0.03 |
| Sertraline | 100mg | £0.04 |
| Sertraline | 150mg | £0.50 |
| Sertraline | 200mg | £0.66 |
| Sertraline | 25mg | £0.32 |
| Sitagliptin | 100mg | £0.06 |
| Tiotropium | 2.5micrograms | £3.83 |
| Spironolactone | 100mg | £0.11 |
| Sumatriptan | 50mg | £0.14 |
| Tiotropium | 18micrograms | £1.12 |
| Trazodone hydrochloride | 100mg | £0.03 |
| Trazodone hydrochloride | 50mg | £0.28 |
| Trimbow | 87micrograms | £0.37 |
| Venlafaxine | 75mg | £0.06 |
| Venlafaxine | 150mg | £0.67 |
| Venlafaxine | 300mg | £1.38 |
| Venlafaxine | 37.5mg | £0.03 |
| Venlafaxine | 225mg | £1.12 |
| Vortioxetine | 10mg | £0.99 |
| Vortioxetine | 20mg | £0.99 |
| Xenidate | 18mg | £0.52 |
| Xenidate | 36mg | £0.71 |
| Zolpidem | 5mg | £0.05 |

**Table A4. Trajectory of costs for COS-P and TAU (NHS and private), based on observed data**

|  | **Baseline** | | **3 months** | | **7 months** | | **12 months** | |
| --- | --- | --- | --- | --- | --- | --- | --- | --- |
|  | **TAU (n = 117)** | **COSI (n = 225)** | **TAU (n = 94)** | **COSI (n = 182)** | **TAU (n = 88)** | **COSI (n = 149)** | **TAU (n = 79)** | **COSI (n = 150)** |
|  | **Cost (£) Mean (SD)** | **Cost (£) Mean (SD)** | **Cost (£) Mean (SD)** | **Cost (£) Mean (SD)** | **Cost (£) Mean (SD)** | **Cost (£) Mean (SD)** | **Cost (£) Mean (SD)** | **Cost (£) Mean (SD)** |
| **GP** |  |  |  |  |  |  |  |  |
| NHS | 64.50 (76.03) | 60.98 (76.75) | 102.69 (101.89) | 82.65 (109.94) | 79.07 (83.54) | 79.26 (96.09) | 80.63 (93.51) | 86.24 (118.90) |
| Private | 0.84 (9.06) | 0.22 (3.27) | 0.00 (0.00) | 1.62 (21.79) | 0.00 (0.00) | 0.66 (5.66) | 0.00 (0.00) | 1.63 (20.00) |
| Total | 65.33 (75.86) | 61.20 (76.65) | 102.69 (101.89) | 84.27 (110.87) | 79.07 (83.54) | 79.91 (95.71) | 80.63 (93.51) | 87.87 (119.39) |
| **Community/District nurse** |  |  |  |  |  |  |  |  |
| NHS | 1.66 (9.71) | 1.34 (7.80) | 1.04 (5.49) | 1.41 (5.87) | 0.73 (3.89) | 0.14 (1.75) | 2.16 (12.71) | 0.85 (5.73) |
| Private | 0.00 (0.00) | 0.00 (0.00) | 0.00 (0.00) | 0.00 (0.00) | 0.00 (0.00) | 0.00 (0.00) | 0.00 (0.00) | 0.00 (0.00) |
| Total | 1.66 (9.71) | 1.34 (7.80) | 1.04 (5.49) | 1.41 (5.87) | 0.73 (3.89) | 0.14 (1.75) | 2.16 (12.71) | 0.85 (5.73) |
| **A&E** |  |  |  |  |  |  |  |  |
| NHS | 43.08 (150.96) | 46.76 (205.60) | 67.87 (212.99) | 73.80 (212.99) | 62.76 (164.39) | 86.49 (187.08) | 66.58 (136.26) | 110.46 (240.45) |
| Private | 0.00 (0.00) | 0.00 (0.00) | 0.00 (0.00) | 0.00 (0.00) | 0.00 (0.00) | 0.00 (0.00) | 0.00 (0.00) | 0.00 (0.00) |
| Total | 43.08 (150.96) | 46.76 (205.60) | 67.87 (212.99) | 73.80 (212.99) | 62.76 (164.39) | 86.49 (187.08) | 66.58 (136.26) | 110.46 (240.45) |
| **Walk-in center** |  |  |  |  |  |  |  |  |
| NHS | 1.38 (10.46) | 3.23 (20.54) | 6.96 (25.58) | 7.91 (25.38) | 8.18 (27.23) | 8.59 (37.36) | 11.14 (41.97) | 11.73 (35.16) |
| Private | 0.00 (0.00) | 0.00 (0.00) | 0.00 (0.00) | 0.00 (0.00) | 0.00 (0.00) | 0.00 (0.00) | 0.00 (0.00) | 0.00 (0.00) |
| Total | 1.38 (10.46) | 3.23 (20.54) | 6.96 (25.58) | 7.91 (25.38) | 8.18 (27.23) | 8.59 (37.36) | 11.14 (41.97) | 11.73 (35.16) |
| **Acute hospital ward** |  |  |  |  |  |  |  |  |
| NHS | 27.12 (152.29) | 27.13 (261.99) | 13.15 (83.09) | 10.64 (71.16) | 11.00 (103.19) | 9.74 (62.14) | 12.25 (65.98) | 19.36 (95.16) |
| Private | 0.00 (0.00) | 0.00 (0.00) | 0.00 (0.00) | 0.00 (0.00) | 0.00 (0.00) | 0.00 (0.00) | 3.06 (27.23) | 0.00 (0.00) |
| Total | 27.12 (152.29) | 27.13 (261.99) | 13.15 (83.09) | 10.64 (71.16) | 11.00 (103.19) | 9.74 (62.14) | 15.32 (70.84) | 19.36 (95.16) |
| **ICU** |  |  |  |  |  |  |  |  |
| NHS | 0.00 (0.00) | 0.00 (0.00) | 0.00 (0.00) | 0.00 (0.00) | 0.00 (0.00) | 0.00 (0.00) | 10.20 (90.64) | 0.00 (0.00) |
| Private | 0.00 (0.00) | 0.00 (0.00) | 0.00 (0.00) | 0.00 (0.00) | 0.00 (0.00) | 0.00 (0.00) | 0.00 (0.00) | 0.00 (0.00) |
| Total | 0.00 (0.00) | 0.00 (0.00) | 0.00 (0.00) | 0.00 (0.00) | 0.00 (0.00) | 0.00 (0.00) | 10.20 (90.64) | 0.00 (0.00) |
| **Community hospital ward** |  |  |  |  |  |  |  |  |
| NHS | 2.09 (22.47) | 0.00 (0.00) | 0.00 (0.00) | 0.00 (0.00) | 0.00 (0.00) | 0.00 (0.00) | 0.00 (0.00) | 6.45 (79.04) |
| Private | 0.00 (0.00) | 0.00 (0.00) | 0.00 (0.00) | 0.00 (0.00) | 0.00 (0.00) | 0.00 (0.00) | 0.00 (0.00) | 0.00 (0.00) |
| Total | 2.09 (22.47) | 0.00 (0.00) | 0.00 (0.00) | 0.00 (0.00) | 0.00 (0.00) | 0.00 (0.00) | 0.00 (0.00) | 6.45 (79.04) |
| **Psychiatric hospital ward** |  |  |  |  |  |  |  |  |
| NHS | 6.26 (67.41) | 0.00 (0.00) | 0.00 (0.00) | 55.85 (507.59) | 0.00 (0.00) | 0.00 (0.00) | 0.00 (0.00) | 0.00 (0.00) |
| Private | 0.00 (0.00) | 0.00 (0.00) | 0.00 (0.00) | 0.00 (0.00) | 0.00 (0.00) | 0.00 (0.00) | 0.00 (0.00) | 0.00 (0.00) |
| Total | 6.26 (67.41) | 0.00 (0.00) | 0.00 (0.00) | 55.85 (507.59) | 0.00 (0.00) | 0.00 (0.00) | 0.00 (0.00) | 0.00 (0.00) |
| **Outpatient** |  |  |  |  |  |  |  |  |
| NHS | 89.22 (271.75) | 79.71 (329.12) | 166.30 (512.29) | 113.74 (532.94) | 92.05 (395.84) | 67.95 (234.97) | 91.14 (245.73) | 93.00 (321.58) |
| Private | 5.82 (46.55) | 0.00 (0.00) | 0.00 (0.00) | 9.89 (133.42) | 0.00 (0.00) | 10.57 (84.08) | 0.00 (0.00) | 6.00 (51.79) |
| Total | 95.04 (273.81) | 79.71 (329.12) | 166.30 (512.29) | 123.63 (547.33) | 92.05 (395.84) | 78.52 (246.65) | 91.14 (245.73) | 99.00 (324.00) |
| **Day hospital** |  |  |  |  |  |  |  |  |
| NHS | 3.88 (29.42) | 9.08 (61.59) | 0.00 (0.00) | 4.95 (40.66) | 15.34 (122.03) | 0.00 (0.00) | 2.85 (25.31) | 9.00 (51.35) |
| Private | 0.00 (0.00) | 0.00 (0.00) | 0.00 (0.00) | 0.00 (0.00) | 0.00 (0.00) | 0.00 (0.00) | 0.00 (0.00) | 0.00 (0.00) |
| Total | 3.88 (29.42) | 9.08 (61.59) | 0.00 (0.00) | 4.95 (40.66) | 15.34 (122.03) | 0.00 (0.00) | 2.85 (25.31) | 9.00 (51.35) |
| **Community care services** |  |  |  |  |  |  |  |  |
| NHS | 12.13 (82.77) | 8.10 (73.55) | 51.02 (401.06) | 8.74 (64.07) | 0.72 (6.72) | 5.29 (31.59) | 1.33 (8.47) | 37.66 (352.54) |
| Private | 0.00 (0.00) | 0.05 (0.70) | 1.37 (13.14) | 0.00 (0.00) | 0.00 (0.00) | 0.00 (0.00) | 0.00 (0.00) | 0.42 (5.14) |
| Total | 12.13 (82.77) | 8.15 (73.55) | 52.39 (401.10) | 8.74 (64.07) | 0.72 (6.72) | 5.29 (31.59) | 1.33 (8.47) | 38.08 (352.54) |
| **Other services** |  |  |  |  |  |  |  |  |
| NHS | 23.35 (77.73) | 21.94 (78.80) | 21.23 (77.17) | 28.38 (154.68) | 7.88 (36.76) | 20.30 (105.69) | 17.54 (84.97) | 0.00 (0.00) |
| Private | 2.17 (18.45) | 0.84 (9.40) | 0.00 (0.00) | 4.85 (44.16) | 0.00 (0.00) | 0.00 (0.00) | 11.16 (99.23) | 0.00 (0.00) |
| Total | 25.53 (79.25) | 22.78 (79.12) | 21.23 (77.17) | 33.23 (159.99) | 7.88 (36.76) | 20.30 (105.69) | 28.71 (129.11) | 0.00 (0.00) |
| **Medication** | 12.66 (31.43) | 13.80 (51.36) | 11.25 (52.44) | 9.82 (22.40) | 10.75 (38.27) | 8.06 (24.09) | 9.30 (46.13) | 6.27 (13.45) |
| **Total healthcare cost** |  |  |  |  |  |  |  |  |
| NHS | 287.80 (417.61) | 270.35 (501.42) | 437.35 (817.85) | 400.63 (815.13) | 291.64 (169.56) | 289.44 (382.44) | 309.36 (342.97) | 383.83 (711.94) |
| Private | 8.84 (50.51) | 0.83 (9.06) | 1.37 (13.14) | 16.35 (141.71) | 0.91 (8.53) | 11.23 (87.66) | 14.23 (102.56) | 8.05 (67.54) |
| Total | 296.63 (428.63) | 271.18 (501.09) | 438.72 (817.39) | 416.99 (820.32) | 292.55 (469.55) | 300.67 (388.30) | 323.59 (364.06) | 391.89 (711.74) |
| **Productivity loss** | 47.44 (246.75) | 36.18 (231.26) | 16.50 (85.52) | 27.17 (148.64) | 45.16 (169.20) | 40.99 (180.61) | 86.23 (327.50) | 61.14 (162.12) |

*Source: our elaboration using trial data and NHS tariffs, PSSRU and BNF.*

**Table A5 One-way sensitivity analysis**

|  | **Base value** | **Lower value** | **Upper value** | **Incremental NMB (lower value)** | **Incremental NMB (upper value)** |
| --- | --- | --- | --- | --- | --- |
| **Intervention cost (TAU)** | £1,942.11 | £762.00 | £3,767.00 | -£388.85 | -£388.85 |
| **The implementation of COS-P** | £343.10 | £267.80 | £474.00 | -£310.11 | -£516.31 |
| **Health service cost (TAU)** | £4,361.43 | £4,233.38 | £4,489.48 | -£513.46 | -£257.36 |
| **Health service cost (COS-P)** | £4,198.90 | £4,126.28 | £4,271.52 | -£312.78 | -£458.02 |
| **QALY (TAU)** | 0.6799 | 0.6747 | 0.6851 | -£229.63 | -£541.18 |
| **QALY (COS-P)** | 0.6731 | 0.6697 | 0.6765 | -£448.10 | -£282.70 |

In the one-way sensitivity analysis, we illustrated how variation in each parameter affects the incremental NMB at the upper willingness-to-pay threshold (£30,000 per QALY gained) recommended by NICE. The lower and upper values of the intervention cost in the TAU arm were based on data provided by each site. For the implementation cost of COS-P, we varied the staff cost for co-facilitator between Band 3 and Band 8. For the health service cost and QALYs in both arms, we derived 95% confidence intervals from the imputed datasets to obtain the possible lower and upper values.
