# Supplementary material for: Cost-utility analysis of the Circle of Security-Parenting programme to reduce perinatal psychopathology in birthing parents in England
Source: BMJ Open. 2026 Feb 23;16(2):e105124. doi: 10.1136/bmjopen-2025-105124 (PMC12931550; doi:10.1136/bmjopen-2025-105124)
Supplement: Supplementary file 2 [file bmjopen-2025-105124.reviewer_comments.pdf]

PEER REVIEW HISTORY

BMJ Open publishes all reviews undertaken for accepted manuscripts. Reviewers are asked to complete a checklist review form and are provided with free text boxes to elaborate on their assessment. These free text comments are reproduced below.

ARTICLE DETAILS

Title (Provisional)

Cost-utility analysis of the Circle of Security-Parenting programme to reduce perinatal psychopathology in birthing parents in England

Authors

Wang, Jiunn; Rosan, Camilla; Alyousefi-van Dijk, Kim; Darwin, Zoe; Babalis, Daphne; Cornelius, Victoria; Waddingham, Ed; Richards, Lani; Pilling, Stephen; Fearon, Pasco; Fonagy, Peter; Pizzo, Elena

---

VERSION 1 - REVIEW

**Reviewer**	**1**
**Name**	**Ride, Jemimah**
**Affiliation**	**Monash University, Centre for Health Economics**
**Date**	**06-Aug-2025**
**COI**	**None**

This study is an economic evaluation alongside a randomised controlled trial. The authors examined the cost-effectiveness of the Circle of Security-Parenting group (COS-P), a perinatal group intervention, provided in addition to the usual National Health Service (NHS) perinatal mental health services, compared to usual NHS perinatal mental health services alone (treatment as usual), for birthing parents in England. The authors, using trial data, estimated the incremental costs and outcomes, the incremental cost effectiveness ratio, and net monetary benefit to assess the cost-effectiveness from both NHS and societal perspectives. The authors found that the intervention is unlikely to be cost effective within the UK NHS perinatal mental health services context.

This study provides valuable insights into the value for money of the intervention to provide information for UK policy and decision makers when making informed choices about the effective use of public health resources. The manuscript is clear, the authors used appropriate methods to answer the research questions, and the conclusions are mostly in line with the results.

Our comments and suggestions are outlined below.

Abstract

• Abstract's results section reports some numbers that do not match the main body of the paper: Numbers stated as having complete economic data appear to mismatch with later information in results. The authors also seem to report results from a complete case analysis (implying that to be the main analysis), while the body of the paper did not report these numbers, and instead reported base results that involved imputation of missing data.

• Abstract includes unspecified acronyms.

• Methods related to sensitivity analysis could be briefly described.

• Results and conclusions seem to over-interpret the statistically insignificant findings.

Introduction

• Bottom paragraph of page 1 has two different styles of referencing

Methods

• Clearer characterisation of the sample would be helpful. Were participants all recruited via perinatal mental health services, as implied, and if so, what does this mean about their clinical/mental health conditions upon entry? Could it be inferred that this sample were all people with a perinatal mental health condition, or some level of elevated symptoms, or just at high risk? This is vital to help the reader understand the evidence generated in the trial.

• Related to the above, the description of the TAU condition could be clearer apart from just listing this in the table. Perhaps with a clearer understanding of who the sample are, their treatment will make more sense, but at the moment it isn't very easy to understand to what extent TAU is likely to differ between intervention and control settings and whether differences in TAU are likely to be contributing to the results.

• The authors stated they estimated TAU costs using data from 6 representative cites, but did not further elaborate on why were the 6 sites chosen and in what ways they were 'representative'.

• It would be good to provide a justification of retrospective resource use data collection.

• The authors stated they estimated both ICERs and NMBs, what is the rationale for including both analyses? The NMB does not seem to add much to the implications, and could be left out to make the interpretation of results more straightforward.

• Missing data: The authors generated five imputed datasets. This number might be too low given the missing data —12-month completion rates of 64.2% for the COS-P group and 48.0% for the TAU group. Several practice guidelines recommend that number of imputed datasets should roughly correspond to the percentage of incomplete cases.

(references:

1. White, I. R., Royston, P., & Wood, A. M. (2011). Multiple imputation using chained equations: Issues and guidance for practice. Statistics in Medicine, 30(4), 377–399. https://doi.org/10.1002/sim.4067

2. Faria, R., Gomes, M., Epstein, D., & White, I. R. (2014). A Guide to Handling Missing Data in Cost-Effectiveness Analysis Conducted Within Randomised Controlled Trials. PharmacoEconomics, 32(12), 1157–1170. https://doi.org/10.1007/s40273-014-0193-3)

• Probabilistic sensitivity analysis: The authors mention assigning statistical distributions to model parameters and conducting bootstrap replications as part of a probabilistic sensitivity analysis. However, they do not specify which parameters were included, the types of distributions assigned, or the degree of variance assumed. These omissions leave the underlying assumptions unclear and limit the interpretability of the results.

Results

• Baseline table: Although baseline costs and outcomes are presented later in the results section alongside other timepoints, the paper would benefit from a baseline table earlier in the results. A table summarising key demographic characteristics, baseline costs, and outcomes across groups would show whether statistically significant differences existed at baseline. This would also support the authors' decision not to adjust for baseline costs or outcomes in their analysis.

• Statements of results don't seem to reflect the non-significance of differences. E.g. it seems to overstate the finding as "generated minimal cost savings" when the difference is £1.63 and highly non-significant.

• Table information and formatting:

Tables 5 and 7, and similarly tables 6 and 8 had the same title. It would be easier to distinguish, and interpret if the titles clearly differentiated them. Additionally, the authors could include the key statistical methods used to derive the estimates in the table footers to improve interpretability.

Key outputs such as the ICER and NMB should be presented with confidence intervals.

• Sensitivity analysis results confirmed main analysis results, however, do not provide additional insight into key parameter uncertainties. One way or scenario analysis might be a better way of characterising the sources of uncertainty and their impact on the results. For example, testing alternative assumptions such as a higher COS-P cost estimate (which the authors suspect), varying TAU costs, and exploring different time horizons could help clarify how these uncertainties and assumptions impact the final ICER.

Discussion

• Similar to the results section, the authors appear to overstate the findings by drawing attention to highly uncertain point estimates, placing more weight on, and extracting meaning from, near-zero point estimates rather than the high degree of non-significance.

For example, “Our economic evaluation indicated marginal NHS cost savings associated with COS-P alongside TAU compared to TAU alone” (page 18, lines 10–12)

• May be worth commenting on the limitations of using the EQ-5D to capture mental health impacts on quality of life

• The multicentre nature of the trial may well be an advantage, but it also seems to bring a lot of uncertainty arising from heterogeneity at different centres in terms of TAU.

• As per comment in results, one way or scenario analysis results might have provided further insights for discussion.

**Reviewer**	**2**
**Name**	**Pisavadia, Kalpa**
**Affiliation**	**Bangor University, School of Medical and Health Sciences**
**Date**	**11-Oct-2025**
**COI**	**None**

The study addresses a highly relevant question in perinatal mental health services and makes a valuable contribution to the evidence base by presenting the first cost-utility analysis of the Circle of Security-Parenting (COS-P) programme within a UK NHS context. The paper is clearly written, methodologically robust, and follows recognised standards for trial-based economic evaluation.

I have a few suggestions that may help strengthen the manuscript further:

1. he estimated cost of COS-P delivery (£343) appears low given staffing bands and session requirements. Please clarify assumptions and, if possible, include a sensitivity analysis using higher cost scenarios to test the robustness of results.

2. EQ-5D-5L may have limited sensitivity to change in this population. A brief discussion of alternative or complementary outcome measures (e.g., mental health–specific utility measures or well-being scales) would be valuable.

3. The discussion could further explore implications for clinical practice, commissioning, and future research including whether COS-P might still be valuable for specific subgroups or if service redesign (e.g., delivery by lower-band staff) could improve cost-effectiveness.

VERSION 1 - AUTHOR RESPONSE

Reviewer: 1
Dr. Jemimah Ride, Monash University
Comments to the Author:
This study is an economic evaluation alongside a randomised controlled trial. The

authors examined the cost-effectiveness of the Circle of Security-Parenting group (COS-P), a perinatal group intervention, provided in addition to the usual National Health Service (NHS) perinatal mental health services, compared to usual NHS perinatal mental health services alone (treatment as usual), for birthing parents in England. The authors, using trial data, estimated the incremental costs and outcomes, the incremental cost effectiveness ratio, and net monetary benefit to assess the cost-effectiveness from both NHS and societal perspectives. The authors found that the intervention is unlikely to be cost effective within the UK NHS perinatal mental health services context.
This study provides valuable insights into the value for money of the intervention to provide information for UK policy and decision makers when making informed choices about the effective use of public health resources. The manuscript is clear, the authors used appropriate methods to answer the research questions, and the conclusions are mostly in line with the results.
Our comments and suggestions are outlined below.

Abstract

• Abstract's results section reports some numbers that do not match the main body of the paper: Numbers stated as having complete economic data appear to mismatch with later information in results. The authors also seem to report results from a complete case analysis (implying that to be the main analysis), while the body of the paper did not report these numbers, and instead reported base results that involved imputation of missing data.

Response: Thank you. We have checked and ensured that there is no discrepancy between the abstract and the main text.

• Abstract includes unspecified acronyms.

Response: we have addressed this now including terms in full followed by acronym.

• Methods related to sensitivity analysis could be briefly described.

Response: Thank you for your comment. We further explained that the sensitivity analysis conducted was the probabilistic sensitivity analysis based on Monte Carlo simulation in the abstract. ***"Probabilistic sensitivity analyses based on Monte Carlo simulation were conducted to assess robustness."***

• Results and conclusions seem to over-interpret the statistically insignificant findings.

Response: We have now extended the conclusion section in the abstract to address the uncertainty around our findings.

Introduction

• Bottom paragraph of page 1 has two different styles of referencing.

Response: we have now amended this.

Methods

• Clearer characterisation of the sample would be helpful. Were participants all recruited via perinatal mental health services, as implied, and if so, what does this mean about their clinical/mental health conditions upon entry? Could it be inferred that this sample were all people with a perinatal mental health condition, or some level of elevated symptoms, or just at high risk? This is vital to help the reader understand the evidence generated in the trial.

Response: thank you for this we added a statement to make this more clear. As stated in the main trial paper *" Participants were birthing parents receiving care from participating community perinatal mental health services. Eligibility criteria included clinical-level psychopathology (defined by an average Clinical Outcomes in Routine Evaluation–Outcome Measure (CORE–OM) score of 1·1 or more and bonding difficulties indicated by a total Postpartum Bonding Questionnaire (PBQ) score of 12 or more)."*

• Related to the above, the description of the TAU condition could be clearer apart from just listing this in the table. Perhaps with a clearer understanding of who the sample are, their treatment will make more sense, but at the moment it isn't very easy to understand to what extent TAU is likely to differ between intervention and control settings and whether differences in TAU are likely to be contributing to the results.

Response: thank you for this we added a statement to make this more clear. "TAU at each of the ten recruitment sites remained unchanged by participants' involvement in the trial and was consistent across study groups. TAU was based on a national service specification, providing multidisciplinary, needs-based care, including pharmacological interventions, parent–infant relational interventions, psychological therapies, and psychosocial support (NHS England

The perinatal mental health care pathways (National Health Service England, 2018). "

• The authors stated they estimated TAU costs using data from 6 representative cites,

but did not further elaborate on why were the 6 sites chosen and in what ways they were 'representative'.

Response: Thank you for your comments. As perinatal mental health care pathways differ across patients, it is challenging to define a TAU should be. To estimate this, we sent questionnaires to all sites to understand the potential standard of care provided. We received responses from six sites. We cross-checked these against our other sites to ensure they were representative in terms of workforce capacity and delivery taxonomies, geographical distribution across regions, a mix of urban and rural areas, and services serving populations with a range of SES/deprivation scores.

We have included this information in the subsection "costs of intervention and comparator" under the section "Resource use and costs".

• It would be good to provide a justification of retrospective resource use data collection.

Response: Thank you for highlighting this. I guess the reviewer refer to TAU data, as the other data are collected retrospectively as per study protocol. We always planned to map TAU between coproduced patient report and service report - to verify and triangulate quality and completion. However - like many others have reported in the past - patient report was poor quality and there was some major discrepancies and so we chose to bespoke retrospective report to get as much detailed and high quality data as possible from a representative sample of services.

This has now been added to the manuscript for clarity. "*We preferred to get a bespoke retrospective report of the services provided to ensure that more detailed and high quality data could be collected by a representative sample of services.* "

• The authors stated they estimated both ICERs and NMBs, what is the rationale for including both analyses? The NMB does not seem to add much to the implications, and could be left out to make the interpretation of results more straightforward.

Response: Thank you for this valuable comment. We report both ICERs and NMBs in line with recommendations from the National Institute of Health and Care Excellence (NICE) reporting guidelines in the UK, which encourages the use of both to facilitate decision-making under uncertainty.

• Missing data: The authors generated five imputed datasets. This number might be too low given the missing data —12-month completion rates of 64.2% for the COS-P group

and 48.0% for the TAU group. Several practice guidelines recommend that number of imputed datasets should roughly correspond to the percentage of incomplete cases. (references:
1. White, I. R., Royston, P., & Wood, A. M. (2011). Multiple imputation using chained equations: Issues and guidance for practice. Statistics in Medicine, 30(4), 377–399. https://doi.org/10.1002/sim.4067
2. Faria, R., Gomes, M., Epstein, D., & White, I. R. (2014). A Guide to Handling Missing Data in Cost-Effectiveness Analysis Conducted Within Randomised Controlled Trials. PharmacoEconomics, 32(12), 1157–1170. https://doi.org/10.1007/s40273-014-0193-3)

Response: Thank you. We have increased the number of imputations to 50 in proportion to the percentage of incomplete cases. Updated description can be found in the "missing data" subsection under the "materials and methods" section. All tables and figures have been updated following this change. References have been updated.

• Probabilistic sensitivity analysis: The authors mention assigning statistical distributions to model parameters and conducting bootstrap replications as part of a probabilistic sensitivity analysis. However, they do not specify which parameters were included, the types of distributions assigned, or the degree of variance assumed. These omissions leave the underlying assumptions unclear and limit the interpretability of the results.

Response: Thank you. We have now explained what distributions and parameters were used in the subsection of "sensitivity analysis". Under the "materials and methods" section. It now reads as "Probabilistic sensitivity analysis was conducted to evaluate robustness by assigning statistical distributions to model parameters: Gamma distributions were used for costs, and Beta distributions for utilities. Parameters in the distributions were based on the statistics such as means and standard deviations derived from the imputed data. Uncertainty was captured using bootstrapping with 1,000 replicates per imputed dataset, and estimates were combined using Rubin's rule".

Results

• Baseline table: Although baseline costs and outcomes are presented later in the results section alongside other timepoints, the paper would benefit from a baseline table earlier in the results. A table summarising key demographic characteristics, baseline costs, and outcomes across groups would show whether statistically significant differences existed at baseline. This would also support the authors' decision not to adjust for baseline costs or outcomes in their analysis.

Response: Thank you. We have now included a table for baseline characteristics, costs and outcomes (Table 3).

• Statements of results don't seem to reflect the non-significance of differences. E.g. it seems to overstate the finding as "generated minimal cost savings" when the difference is £1.63 and highly non-significant.

Response: As we changed the number of imputation, the estimates were slightly different from the last version. There is no cost savings based on the current imputed results. We also recognised that the potential overstatement, so we were careful about the interpretation in this version.

• Table information and formatting:
Tables 5 and 7, and similarly tables 6 and 8 had the same title. It would be easier to distinguish, and interpret if the titles clearly differentiated them. Additionally, the authors could include the key statistical methods used to derive the estimates in the table footers to improve interpretability.

Response: We have now revised the titles of these tables (currently Tables 6, 7, 8, and 9) to make them easier to distinguish. We have also added footnotes to clarify the key methods used to derive the estimates.

Key outputs such as the ICER and NMB should be presented with confidence intervals.

Response: Thank you for your comment. We acknowledge the high uncertainty around the incremental costs and QALYs. As shown in Figures 1 and 2, the estimates are widely dispersed on the cost-effectiveness plane. Following Briggs et al (2002), we believe that confidence intervals for ICER may be of limited value useful, particularly in our case where both incremental costs and QALYs could be either negative or positive, and the incremental QALY is very small. To better capture the uncertainty, we have therefore the cost-effectiveness planes in Figures 1 and 2 and cost-effectiveness acceptability curve instead.

Briggs, A.H., O'Brien, B.J. and Blackhouse, G., 2002. Thinking outside the box: recent advances in the analysis and presentation of uncertainty in cost-effectiveness studies. *Annual review of public health*, *23*(1), pp.377-401.

• Sensitivity analysis results confirmed main analysis results, however, do not provide additional insight into key parameter uncertainties. One way or scenario analysis might be a better way of characterising the sources of uncertainty and their impact on the results. For example, testing alternative assumptions such as a higher COS-P cost

estimate (which the authors suspect), varying TAU costs, and exploring different time horizons could help clarify how these uncertainties and assumptions impact the final ICER.

Response: Thank you. We have included a one-way sensitivity analysis in the supplementary material (Table A3). By focusing on the incremental NMB, the one-way sensitivity analysis suggests that COS-P is still not cost-effective compared with TAU even when we vary parameters within reasonable ranges.

Discussion

• Similar to the results section, the authors appear to overstate the findings by drawing attention to highly uncertain point estimates, placing more weight on, and extracting meaning from, near-zero point estimates rather than the high degree of non-significance. For example, "Our economic evaluation indicated marginal NHS cost savings associated with COS-P alongside TAU compared to TAU alone" (page 18, lines 10–12)

Response: this has now been addressed, also given the different results from new imputation analysis.

• May be worth commenting on the limitations of using the EQ-5D to capture mental health impacts on quality of life

Response: thank you for this. We have now included a sentence in the discussion.
"Fifth, the EQ-5D questionnaire might not be the best instrument to capture postnatal mental health impacts on quality of life, because it doesn't directly assess aspects such as fatigue, cognitive issues, social isolation, spirituality, and other emotional states beyond "anxiety and depression" that are relevant to birthing parents."

• The multicentre nature of the trial may well be an advantage, but it also seems to bring a lot of uncertainty arising from heterogeneity at different centres in terms of TAU.

Response: we agree, this has now been mentioned in the paper, both in methods and discussion.

• As per comment in results, one way or scenario analysis results might have provided further insights for discussion.

Response: Thank you. We have included an one-way sensitivity analysis in the supplementary materials (Table A3).

Reviewer: 2
Miss Kalpa Pisavadia, Bangor University
Comments to the Author:
The study addresses a highly relevant question in perinatal mental health services and makes a valuable contribution to the evidence base by presenting the first cost-utility analysis of the Circle of Security-Parenting (COS-P) programme within a UK NHS context. The paper is clearly written, methodologically robust, and follows recognised standards for trial-based economic evaluation.

I have a few suggestions that may help strengthen the manuscript further:

1. he estimated cost of COS-P delivery (£343) appears low given staffing bands and session requirements. Please clarify assumptions and, if possible, include a sensitivity analysis using higher cost scenarios to test the robustness of results.

Response: Thank you. We have now addressed this including a one-way sensitivity analysis in Table 3A in the supplementary materials.

2. EQ-5D-5L may have limited sensitivity to change in this population. A brief discussion of alternative or complementary outcome measures (e.g., mental health–specific utility measures or well-being scales) would be valuable.

Response: thanks, we have now addressed this in the discussion including a paragraph "*Fifth, the EQ-5D-5L questionnaire might not be the best instrument to capture postnatal mental health impacts on quality of life, because it doesn't directly assess aspects such as fatigue, cognitive issues, social isolation, spirituality, and other emotional states beyond "anxiety and depression" that are relevant to birthing parents, as well as it has limited sensitivity to change in this population.* "

3. The discussion could further explore implications for clinical practice, commissioning, and future research including whether COS-P might still be valuable for specific subgroups or if service redesign (e.g., delivery by lower-band staff) could improve cost-effectiveness.

Response: Thank you for this. At the time we submitted this work the main trial paper was not published yet. Now it is, and we can be more specific. We added the following in the discussion "In addition to the cost-effectiveness results reported here, the COSI trial has found no additional clinical benefit from COS-P as compared to TAU alone (Rosan et al., 2025). Therefore, we do not recommend the use of COS-P in NHS Perinatal Mental Health Services in the format used here."
